# *CDApps*: integrated software for experimental planning and data processing at beamline B23, Diamond Light Source

**DOI:** 10.1107/S1600577514028161

**Published:** 2015-01-31

**Authors:** Rohanah Hussain, Kristian Benning, Tamas Javorfi, Edoardo Longo, Timothy R. Rudd, Bill Pulford, Giuliano Siligardi

**Affiliations:** a Diamond Light Source Ltd, Diamond House, Harwell Science and Innovation Campus, Didcot, Oxfordshire OX11 0DE, UK

**Keywords:** synchrotron radiation circular dichroism (SRCD), vacuum-ultraviolet (VUV), secondary structure, protein, denaturation, *CDApps*, integrated software

## Abstract

The B23 Circular Dichroism beamline at Diamond Light Source has been operational since 2009 and has seen visits from more than 200 user groups, who have generated large amounts of data. Based on the experience of overseeing the users’ progress at B23, four key areas requiring the most assistance are identified: planning of experiments and note-keeping; designing titration experiments; processing and analysis of the collected data; and production of experimental reports. To streamline these processes an integrated software package has been developed and made available for the users. The subsequent article summarizes the main features of the software.

## Abstract 2

*CDApps* software at Diamond B23 SRCD beamline is presented.

## 1. Introduction   

Synchrotron radiation circular dichroism (SRCD) is a well established technique in structural biology. Circular dichroism (CD) is the spectroscopic technique of choice to obtain low-resolution structural information of biopolymers such as proteins, nucleic acids, carbo­hydrates, lipids and small organic molecules (drugs) (Fasman, 1996 ▶; Berova *et al.*, 2000 ▶). Diamond Light Source’s first UV-VIS beamline dedicated to circular dichroism, B23 (Hussain *et al.*, 2008 ▶, 2012 ▶; Jávorfi *et al.*, 2010 ▶; Siligardi *et al.*, 2010 ▶), has been operational since 2009.

In keeping with the philosophy of other synchrotron facilities a limited amount of time is allocated to each user group to perform their projects; at B23 this is usually six to nine shifts (where a shift is a block of 8 h). This time constraint puts pressure on users to shift the emphasis towards data collection and as a consequence the subsequent data analysis often lags behind. The collection of a large number of files that require the same type of processing and analysis raised the need for the development of a computer program capable of performing batch processing.

A typical CD experiment entails the recording of spectral information (amplitude *versus* wavelength) or kinetic data (amplitude *versus* time) of the sample while other parameters (such as temperature, pH *etc.*) are kept constant or changed in a controlled fashion. With all of the modern spectrometers available on the market, instrument control and data collection is performed by a PC, which greatly simplifies the experimental procedure. All manufacturers provide their own software for these tasks; usually these are also equipped, to a greater or lesser extent, with extra functionality to aid data processing/analysis. Most of the time this is adequate to process the results of an experiment; some of them even have the ability to generate a report file. However, they usually cannot cope with batch processing of multiple data files or collating the analysis of different types of experiments.

The processing and analysis of the experimental data has always been the limiting factor in the process of planning further measurements or compiling a report after a beamline visit. To address this issue, *CDApps* was created to streamline this important part of experimental visits to B23. The graphical user interface is meant to be user friendly and the design took into account the comments and requirements from our beta testers. Here we described the *CDApps* software v4.0, which has been released to our users (January 2014).

Between October 2012 and March 2014 there had been two releases of *CDApps* to our users. Two data-processing workshops were carried out, one in April 2013 and the other in November 2013, with 12 of our current or potential users at each occasion. The workshops were divided into two sessions, with six tutors in each session providing a one-to-one introduction to the usage of *CDApps*. Remote access was also made available where the users could see all their data files from previous visits and process their data using *CDApps* from their home institute. These experiences were evaluated and their outcome assisted us in defining the software.

## 2. Beamline software overview   

Previous experience had shown that CD experiments would benefit considerably from:

(i) Increased automation.

(ii) Direct control of more experimental hardware.

(iii) Remote access allowing users to pre-plan their experiments.

(iv) Integrating protein secondary structure estimation.

This has now been delivered by *CDApps*.

*CDApps* is a Visual Basic .NET application that was specifically designed to help B23 beamline users to plan their CD experiments and to streamline their data processing. As it has evolved from an earlier Microsoft Excel VBA macro set, it still makes extensive use of the graphing and spreadsheet handling capabilities of Excel. Hence, in order to run *CDApps* it is a prerequisite to have Microsoft Excel installed on the computer. The users’ logbook, raw data files from an experiment, processed data and the analysis results are all stored in various worksheets of a single Excel file and can also be opened later on any other computer without running *CDApps*. This also includes a macro-enabled index sheet to take you to your chosen sheet. *CDApps* integrates with Diamond’s Oracle database so that when logged-in users load *CDApps* it displays all their previous and upcoming visits including the visit dates, project reference, visit description and the proposal abstract. However, this is not an essential component of *CDApps*; the data folder can be selected manually and the analysis package can also be used without the link to the project database. Since the primary goal was to use *CDApps* in connection with the beamline end-station, run using *Olis Global Works* software, it can directly import native (binary) OLIS data files (*.ols), which are then converted to Excel worksheets. However, practically all data formats that can be imported to an Excel worksheet can be treated by *CDApps*; import functions for tab- or comma-separated values (*.txt or *.csv files) are also included.

*CDApps* has been coded and compiled using Microsoft’s Visual Studio 2010 to run on Windows XP or Windows 7/8 machines. *CDApps* makes use of Extreme Optimization^TM^’s numeric library for curve fittings in some of the analyses.

## 3. Beamline experimental protocol   

**Figure 1:**
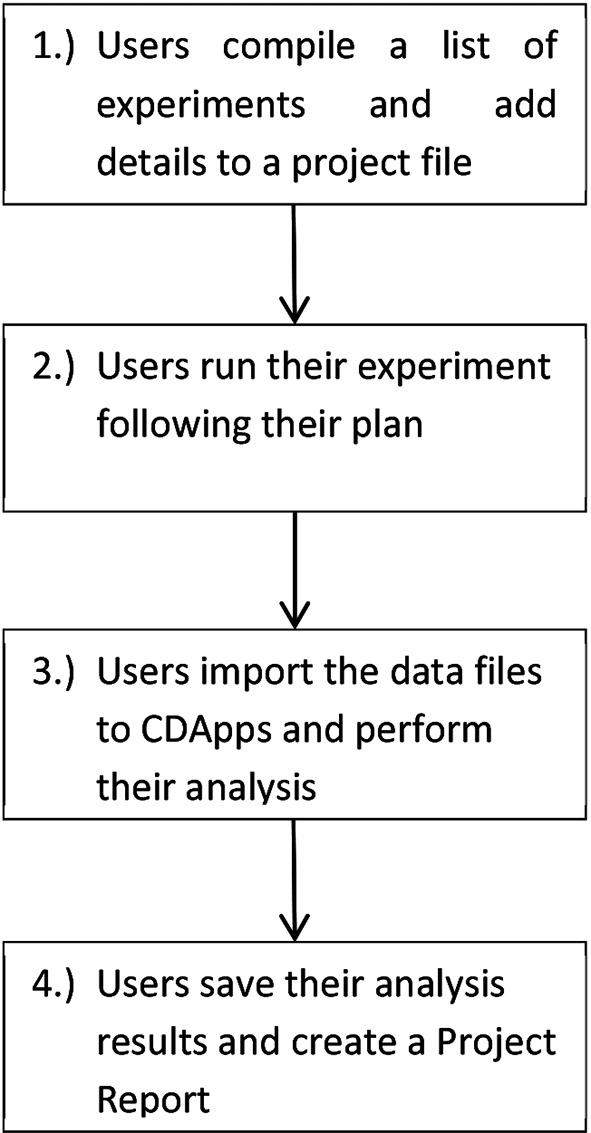
Flow chart of the experimental protocol.

In general, the users should go through a sequence of processes in order to use their beam time efficiently and to be able to perform a successful experiment. These steps can be summarized in the flow-chart shown in Fig. 1 ▶. *CDApps* was designed with the objective to help the users with all four areas in this process.

### 3.1. Step 1   

At Diamond Light Source the users are supplied with a FED ID that allows them access to the Windows workstations at the beamline (http://www.diamond.ac.uk/Users/UserGuide.html). When they load *CDApps*, recognition of the users’ FED ID allows specific information, such as a list of their previous visits, names and project numbers, to be automatically retrieved from Diamond’s project database. Then the users have the option to choose which type of CD experiments they wish to conduct: *CD Titration* or *CD Measurement*. These two options offer different functionality. In *CD Titration* the users can design a ligand binding experiment: given the sample details (concentration of stock solutions, extinction coefficients) and estimation for the binding constant (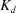) (Siligardi *et al.*, 2002 ▶) the program can calculate the sample volumes to be used and the working concentrations. There are also some indications if the desired working concentrations can be achieved within the volume of the cuvettes used for the experiment or if more concentrated stock solutions should be prepared. Following the *CD Measurement* option the users can create a list of experiments that fall into three basic categories:

(i) Repeated scans (1 to *n* scans), where the spectra are handled as one and averaged to provide the final spectrum.

(ii) UV denaturation experiment. In this circumstance each spectra is handled separately allowing the change in CD intensity as a function of the number of scans (*i.e.* dose) to be monitored.

(iii) Thermal melt, where repeated scans are recorded as a function of sample temperature. Choosing this last option the users have the possibility to create a script file, containing the temperature values and incubation times that can be imported to the beamline endstation software for execution.

The experimental planning can be performed using *CDApps* at the beamline or *via* remote access before the allotted beamline visit.

### 3.2. Step 2   

In the second step of the process the users open their pre-compiled list of experiments and record the data according to their plan using beamline control software. At the moment of writing this manuscript, *CDApps* does not offer extra functionality here; however, additional features are currently being tested that will allow the users to execute the data collection routines directly from *CDApps*, permitting greater automation of the experimental process in the future. This will help to eliminate the need for entering certain parameters twice (for *CDApps* and for the beamline control software) and it will automatically assign the data files to the experiments reducing the risk of making errors.

### 3.3. Step 3   

Native OLIS data files (*.ols) can be imported directly; however, other file formats (generated by other spectrometers) need to be converted to ASCII (X–Y) format first (*.csv or *.txt files are accepted). The imported data files then have to be linked to the specific experiments before the analysis. In general, a baseline spectrum (of the experimental buffer or air) needs to be selected as well, which will then be subtracted from the sample files. If the sample details are entered, *CDApps* will automatically convert the CD units from mdeg (recorded instrument units) to differential absorption and extinction coefficient values, Δ*A* and Δ∊, respectively, creating separate graphs for each of the converted datasets. Depending on the type of experiment, the software will try to fit a binding curve with the titration data, and calculate the binding constant, assuming a single binding site, or fit a melting curve at any selected wavelength, based on the Boltzmann algorithm, and give an estimation for the melting temperature (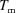).

The software also offers an option to determine the secondary structure composition of the samples (proteins and peptides), using three different algorithms (CONTINLL, CDSSTR or SELCON3) (Provencher & Glockner, 1981 ▶; Van Syokkum *et al.*, 1990 ▶; Sreerama & Woody, 2000 ▶) obtained from OLIS secondary structure estimation library; the users can choose from a list of reference datasets according to the spectral range of their own experimental data and the type of material that is being examined, *e.g.* soluble, membrane or denatured proteins or peptides.

One semi high-throughput option at B23 is the use of a six-cell sample changer that allows automated data collection for up to six samples; in this circumstance *CDApps* gives the users the possibility to split up the data file to up to six sub-sets (depending on the actual number of samples placed in the turret) and treat the resulting sub-sets as individual sample data during the analysis. This allows the users to plan long experiments (possibly to run overnight) without intervention during the measurement.

### 3.4. Step 4   

At the end of the analysis, the results, together with the imported and processed data, and the different graphs can be saved as an Excel file; the file will contain many worksheets and an automatically generated index sheet to help the users to navigate between the different pages. The users can take this file home, where it can be re-opened without the need for *CDApps*, if they would like to change the format of the graphs or copy the analysis results into their preferred software package for further processing. *CDApps* also offers a report-generating function that then can be submitted to Diamond as an end-of-beam-time report. This is a pre-formatted Word document, which contains the user details, the abstract of the project (if it was used in connection with a beamline visit) and any number of graphs that the user selects from the analysis results. It will also add blank paragraphs where the users can insert their discussion, future work, conclusions and references.

## 4. Facility software access   

**Figure 2:**
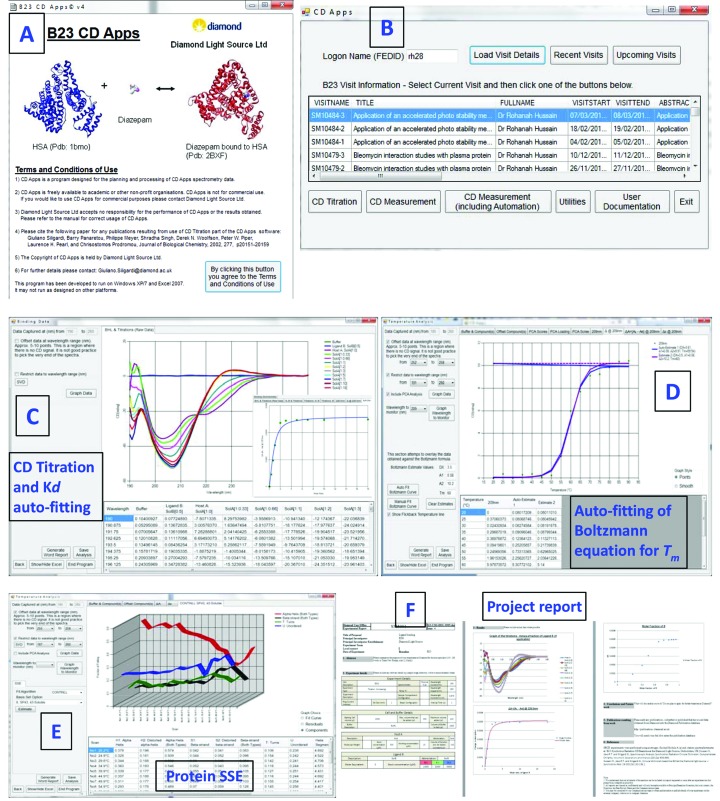
Summary of *CDApps* presentations showing (A) the application page, (B) beam time visit information, (C) CD titration for ligand binding with *K*_*d*_ auto-fitting function, (D) CD measurement for thermal melt with auto-fitting by Boltzmann algorithm for 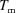 determination, (E) protein secondary structure estimation using CONTINLL, SELCON3 or CDSSTR algorithms for batch processing, (F) project report generated in a Word document.

*CDApps* v4.0 is now available on the B23 beamline workstations for our users. Since it requires a controlled and standardized software environment maintained by Diamond Light Source, it cannot be downloaded and run on the users own computer. However, it can be used outside of the facility remotely after connecting to Diamond’s network. In this case users will have to log-in to https://remote.diamond.ac.uk using their FED ID and password, which is allocated when users register to apply for beam time, and follow the instructions there to connect to a virtual Windows machine. Summary of *CDApps* software utility and graphical presentations is shown in Fig. 2 ▶.

Information on the *CDApps* can be found at the Diamond B23 website (http://www.diamond.ac.uk/Beamlines/Soft-Condensed-Matter/B23/manual/Beamline-software.html). The user manual can be accessed from *CDApps* itself or *via* the Diamond webpage of the beamline (http://confluence.diamond.ac.uk/display/B23Tech/CD+Apps+documentation). A summary of the main features can be found in Table 1 ▶.

A video tutorial is also available on the B23 website, with step-by-step guidance to process the results of a variable-temperature experiment, as an example (http://www.diamond.ac.uk/Beamlines/Soft-Condensed-Matter/B23/manual/Beamline-software/video-tutorials.html#).

## 5. Future development   

Some additional features of the software are at present in beta-testing phase. These are mostly related to additional instrument controls, such as a temperature controller (Quantum or Linkam) or X–Y stage controller, and further integration to the beamline controller software. When fully functional then the users should only use one single platform, *CDApps*, to set up all instrument parameters and experimental conditions and the measurements would be carried out automatically.

## Figures and Tables

**Table 1:** Beamline B23 *CDApps* features

Features available	Notes
Link to the submitted project details	Requires the Oracle client to be installed
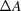 and 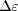 auto-conversion	Based on molecular weight and concentration inputs from the user
Overlay of multiple spectra	Comparative viewing and assessment
Auto and manual fitting of the binding constant ( 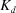 )	Non-linear regression for single binding site
Stoichiometric determination	Indication of single or multiple binding sites
Auto and manual fitting of the melting temperature ( 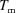 )	Single Boltzmann algorithm
Secondary structure estimation for proteins and peptides	Auto fitting using various reference dataset libraries and three main algorithms: CONTINLL, CDSSTR or SELCON3
Monitor the change in intensity at a single wavelength	Users can pick any wavelength value from a set of spectra to plot the changes in intensity as a function of time, temperature or scan number
Cope with data sets obtained with the six-cell turret	Data set can be split up to subsets belonging to the different samples in the turret and these subsets can be processed individually
Processed data and the analysis results are kept in various worksheets	Data are stored in an Excel file that can also be opened later on any computer
Report generation	Generates a pre-formatted Word document that includes the project number, name of users, abstract from peer-reviewed proposal, experimental details and selected figures; the discussion, conclusion, future work and references sections need to be filled in by the users
