# *CDApps*: integrated software for experimental planning and data processing at beamline B23, Diamond Light Source. Corrigendum

**DOI:** 10.1107/S1600577515007602

**Published:** 2015-04-30

**Authors:** Rohanah Hussain, Kristian Benning, Daniel Myatt, Tamas Javorfi, Edoardo Longo, Timothy R. Rudd, Bill Pulford, Giuliano Siligardi

**Affiliations:** a Diamond Light Source Ltd, Diamond House, Harwell Science and Innovation Campus, Didcot, Oxfordshire OX11 0DE, UK

**Keywords:** synchrotron radiation circular dichroism (SRCD), vacuum-ultraviolet (VUV), secondary structure, protein, denaturation, *CDApps*, integrated software

## Abstract

In the paper by Hussain *et al.* ▶ [(2015), *J. Synchrotron Rad.***22**, 465–468], Daniel Myatt is missing from the list of authors. The complete list of authors should be Rohanah Hussain, Kristian Benning, Daniel Myatt, Tamas Javorfi, Edoardo Longo, Timothy R. Rudd, Bill Pulford and Giuliano Siligardi.

## Abstract 2

A correction is made to the article by Hussain *et al.* [(2015), *J. Synchrotron Rad.***22**, 465–468].

## References

[bb1] Hussain, R., Benning, K., Javorfi, T., Longo, E., Rudd, T. R., Pulford, B. & Siligardi, G. (2015). *J. Synchrotron Rad.* **22**, 465–468. 10.1107/S1600577514028161 PMC4344363 25723950

